# Performance of Rapid Point-of-Care and Laboratory Tests for Acute and Established HIV Infection in San Francisco

**DOI:** 10.1371/journal.pone.0080629

**Published:** 2013-12-12

**Authors:** Christopher D. Pilcher, Brian Louie, Shelley Facente, Sheila Keating, John Hackett, Ana Vallari, Chris Hall, Teri Dowling, Michael P. Busch, Jeffrey D. Klausner, Frederick M. Hecht, Sally Liska, Mark W. Pandori

**Affiliations:** 1 HIV/AIDS Division, University of California San Francisco, San Francisco, California, United States of America; 2 Public Health Laboratory, San Francisco Department of Public Health, San Francisco, California, United States of America; 3 HIV Prevention Section, San Francisco Department of Public Health, San Francisco, California, United States of America; 4 STD Prevention and Control Services Section, San Francisco Department of Public Health, San Francisco, California, United States of America; 5 Blood Systems Research Institute, San Francisco, California, United States of America; 6 Abbott Diagnostics, Abbott Park, Illinois, United States of America; 7 Magnet Community Health Center, San Francisco, California, United States of America; McGill University Health Centre, McGill University, Canada

## Abstract

**Background:**

Current laboratory and point-of-care tests for HIV detect different analytes and use different sample types. Some have fast turnaround times (<1 hour). We investigated how HIV test choice could impact case finding by testing programs.

**Methods:**

We analyzed 21,234 consecutive HIV tests with venous blood obtained by San Francisco HIV testing programs from 2003 to 2008. For a subset, oral fluid (n = 6446) or fingerstick blood (n = 8127) samples were also obtained for rapid testing. In all cases, HIV status was determined using an HIV antibody-plus-RNA test algorithm. We assessed how the screening antibody tests performed individually versus the gold standard of the full algorithm. We then evaluated the potential ability of other tests (including new tests) to detect more cases, by re-testing all specimens that had negative/discrepant antibody results on initial screening.

**Findings:**

The antibody-RNA algorithm identified 58 acute and 703 established HIV infection cases. 1^st^-generation (Vironostika) and 3^rd^-generation (Genetic Systems) immunoassays had 92 and 96 percent sensitivity, respectively. The Oraquick rapid test had clinical sensitivity of only 86 percent on oral fluid samples, but 92 percent on finger-stick blood. Newer 4^th^-generation, antigen-antibody combo rapid immunoassay (ARCHITECT) detected HIV in 87 percent of all the acute cases that had been missed by one of the previous screening assays. A point-of-care 4^th^ generation antigen-antibody combo rapid test (Determine) detected about 54 percent of such acute cases.

**Conclusions:**

Our study suggests that some rapid antibody blood tests will give similar case detection to laboratory antibody tests, but that oral fluid testing greatly reduces ability to detect HIV. New 4^th^-generation combo tests can detect the majority of acute infections detectable by HIV RNA but with rapid results. Using these tests as a primary screening assay in high-risk HIV testing programs could reduce or eliminate the need for HIV RNA testing.

## Introduction

A substantial number of individuals seek (or are referred for) HIV screening at HIV testing sites during the earliest, acute phase of HIV infection, when HIV antibody responses are evolving [1]–[6]. As a result, traditional HIV antibody test screening misses between 1 and 13 percent of cases of HIV infection that are potentially detectable through HIV testing programs ([1]–[6]; reviewed in [7]). Reliably identifying such individuals as HIV-infected is an important public health concern because the potential for sexual transmission is very high [8]–[10], and very early treatment might confer clinical benefits [11].

For testing sites where acute HIV infections may be frequent, the US Association of Public Health Laboratories and CDC [12] support the use of HIV RNA tests as supplemental screening tests. The addition of HIV RNA testing increases both the cost and complexity of HIV testing; while the use of HIV RNA testing has been shown to be highly cost effective when applied in high-incidence populations [13], most laboratories serving high risk populations have not yet adopted this approach. Indeed, many testing programs prefer tests that can give rapid results back to providers and patients.

HIV tests have recently undergone considerable development, and multiple studies have suggested that a high number of acute HIV infections may be detected by the some of the most sensitive new assays that simultaneously detect HIV-1 *gag* p24 antigen (Ag) and antibody (called “4^th^ generation” or “combo” immunoassays) [6], [14]–[16]. In particular, two recently introduced combo tests are capable of providing preliminary, single sample results within one hour: one, the Determine HIV 1/2 Ag-Ab Combo, is a conventional lateral flow rapid test device, while the ARCHITECT HIV Ag/Ab Combo is an immunoassay that uses an automated analyzer and can be used either for analysis of specimens in batch or, in random access mode, for single sample, “rapid results” testing. Also in the last year, one oral fluid test has been approved by the US FDA for home use and is being widely distributed. In this study, we sought to determine how use of these newer tests might perform given their variable ability to detect acute infections. We analyzed unique specimens and data at the San Francisco Department of Public Health, where large programs for targeted HIV antibody-plus-HIV RNA testing have been conducted since 2003. The SFDPH has systematically catalogued all specimens with either false-negative or false-positive HIV antibody or HIV RNA test results. We used these stored data and specimens to assess how new tests might influence the performance of HIV testing programs in San Francisco.

## Methods

This program evaluation was approved by the University of California, San Francisco Committee on Human Subjects Research (UCSF CHR) and was conducted in accordance with the Declaration of Helsinki. Collection of data on HIV testing outcomes and archiving of testing specimens from public testing sites were conducted as part of routine public health practice by the San Francisco Department of Public Health. Review of this study by the UCSF CHR found that the project was therefore exempt from the requirement for obtaining specific informed consent from testing clients.

### Study design and participating “targeted testing” programs

This was a retrospective public health program evaluation involving publicly funded HIV testing programs that 1) provided “targeted” HIV testing (i.e., voluntary testing for clients self-identifying as at risk for HIV infection) in San Francisco, and 2) supplemented first-line HIV antibody testing with HIV RNA testing to exclude the possibility of false-negative antibody test results. The majority of specimens and data for the study were obtained from patients receiving publicly funded HIV voluntary counseling and testing through these programs between 2003 and 2008. Targeted testing programs served the following populations:

- *STD Clinic Population (n = 14,573):* Attendees to the San Francisco City Clinic, a municipal STD clinic, requested or were offered HIV testing. City Clinic clients who were high-risk men who have sex with men (MSM) are described below and are considered separately.
- *High Risk Men who have Sex with Men (MSM) (n = 6661):* Selected MSM meeting criteria for particularly high risk of HIV infection at the San Francisco City Clinic and at two gay-focused community-based health centers (Magnet and AIDS Health Project) were offered initial point-of-care HIV rapid testing along with HIV RNA testing. Criteria for inclusion in this program varied slightly by site but included self-report of unprotected anal intercourse, any intercourse with an HIV positive individual, or having signs or symptoms of another sexually transmitted infection.
- *Non-Occupational Post-Exposure Prophylaxis (nPEP) (n = 989).* Men and women were tested who presented to a City Clinic run program requesting post-exposure prophylaxis with antiretroviral therapy following sex with a person with known or suspected HIV infection. Tests included both baseline and follow-up evaluations.
- *Partner Services (PS) Testing Population (n = 173)*. Sex or needle-sharing partners of patients newly diagnosed with HIV infection were offered voluntary HIV counseling and testing at the San Francisco City Clinic.
- *Sex Worker Population (n = 1110).* Sex workers receive health services including screening for HIV infection at the St. James Infirmary clinic.

In addition to patients from the Department of Health targeted testing programs, we included additional specimens obtained from an academic research-oriented testing program, the UCSF Options Study *(n = 1114; 1998–2008).* Patients were included in this screening program based on suspicion of having acute or recent HIV infection; some had prior positive results available.

### The HIV antibody-HIV RNA testing algorithm

**Figure 1:**
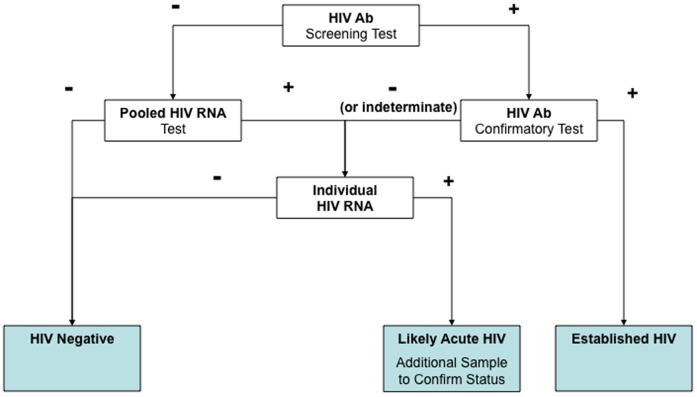
Clinical Testing Algorithm, San Francisco Targeted Testing Programs 2003–2008 (adapted from [6]).

A serial algorithm that included HIV antibody testing for all specimens and HIV RNA testing for all antibody-negative or indeterminate specimens is illustrated in Figure 1.

#### HIV antibody testing

Different types of HIV antibody assays were used by the various programs for initial screening, as indicated in Table 1. The specific totals of specimens tested by each assay are shown in Table 2. The majority of high-risk patients were screened first using a 1^st^ generation HIV antibody immunoassay [Vironostika HIV-1 Microelisa System, bioMérieux Inc., Durham, NC, USA] in the Public Health Laboratory. After 2007, the laboratory used the Genetic Systems HIV ½ Plus O assay [Biorad, Redmond, WA, USA]. Between 2005 and 2008, however, several programs serving *High Risk MSM, nPEP, Partner Services and Sex Worker* populations began providing point-of-care HIV rapid testing [Oraquick Advance Rapid HIV ½ Antibody Test; Orasure Technologies, Bethlehem, PA, USA] for high risk patients, with blood drawn only for completion of RNA testing and/or confirmatory antibody testing in the Public Health Laboratory.

**Table 1:** Identification of Acute (HIV Antibody Negative or Indeterminate) and Established (Confirmed HIV Antibody Positive) HIV Infections by Targeted Testing Programs in San Francisco, 2003–2008.

		HIV Case Identification			Acute HIV Case Identification			Established HIV Case Identification		
Program	Testing	N	n	(% prev)	n	% (of cases)	% (of testers)	n	% (of cases)	% (of testers)
**All DPH Targeted Testing**		21234	761	3.6%	58	7.6%	0.3%	703	92.4%	3.3%
Sex Worker	RT, IA	1110	16	1.4%	0	0.0%	0.0%	16	100.0%	1.4%
nPEP	IA	989	20	2.0%	8	40.0%	0.8%	12	60.0%	1.2%
STD Clinic	IA	13321	350	2.6%	23	6.6%	0.2%	327	93.4%	2.5%
High Risk MSM	RT	5641	355	6.3%	25	7.0%	0.4%	330	93.0%	5.9%
Partner Services	RT, IA	173	20	11.6%	2	10.0%	1.2%	18	90.0%	10.4%
**Acute HIV Screening** *	IA	1114	845	75.9%	90	10.7%	8.1%	755	89.3%	67.8%
**All High Risk Programs** *		22348	1606	7.2%	148	9.2%	0.7%	1458	90.8%	6.5%

= rapid test; IA = immunoassay. RT

Include results from the acute HIV screening program results for years 1998 through 2008.

**Table 2:** Performance Characteristics of Four HIV Antibody Screening Tests Used by San Francisco Targeted Testing Programs, 2003–2008.

Screening Test Used	Patient Tested	Actual Case Identification-n (%)			False+	Actual Performance Estimates-% (95% confidence interval)			
		Acute HIV	Established HIV	All HIV		Sensitivity	Specificity	PPV	NPV
Oraquick Advance (Oral Fluid RT)	6446	0/11 (0.0)	110/116 (94.8)	110/127 (86.6)	5	86.6 (79.4, 92.0)	99.9 (99.8, 100.0)	95.7 (90.1, 98.6)	99.7 (99.6, 99.8)
Oraquick Advance (Fingerstick blood RT)	8127	0/18 (0.0)	226/228 (99.1)	226/246 (91.9)	1	91.9 (87.7, 95.0)	100.0 (99.9, 100.0)	99.6 (97.6, 100.0)	99.7 (99.6, 99.8)
Vironostika HIV-1 Microelisa	2860	0/22 (0.0)	262/262 (100.0)	262/284 (92.3)	0	92.3 (88.5, 95.1)	100.0 (99.9, 100.0)	100.0 (98.6,100.0)	99.2 (98.7, 99.5)
Genetic Systems HIV 1/2 Plus O	3801	3/7 (27.3)	97/97 (100.0)	100/104 (96.2)	0	96.2 (90.4, 98.9)	100.0 (99.9, 100.0)	100.0 (96.4, 100.0)	99.9 (99.7, 100.0)
All Clinical Testing	21234	4/58 (6.9)	694/703 (98.7)	698/761 (91.7)	6	91.7 (89.5, 93.6)	100.0 (99.9, 100.0)	99.1 (98.2, 99.7)	99.7 (99.6, 99.8)

= rapid test; IA = immunoassay. RT

For all programs, specimens that were reactive on initial HIV antibody screening test (regardless of specimen type) underwent confirmatory testing using a plasma specimen with an FDA-approved HIV antibody immuno-fluorescence assay [Flourognost HIV-1 IFA: Waldheim Pharmazeutika GmbH; Vienna, Austria] and/or HIV antibody Western blot [Biorad Genetic Systems HIV-1 WB; Biorad, Redmond, WA, USA].

#### HIV RNA testing

Specimens from the UCSF Options study, who had suspected acute or recent HIV infection, were screened individually for HIV RNA, without pooling, using the AMPLICOR HIV-1 Monitor Test, v1.5 [Roche Molecular Systems, Inc., Branchburg, NJ] or Versant HIV-1 RNA 2.0 [Siemens Medical Solutions Diagnostics, Berkeley, CA]. In the publicly funded testing programs, specimens that were non-reactive on the initial screening assay were pooled (10∶1) for HIV RNA screening as previously described [2], [3] using an HIV RNA test with a lower limit of detection of 75 copies per mL HIV RNA or lower [2003–2007: Versant HIV-1 RNA 3.0 assay; Siemens; 2007–2008: Abbott m2000 RealTi*m*e HIV-1 Assay, Abbott Molecular Inc.; Des Plaines, IL, USA]. After pooled testing, specimens from pools that were reactive for HIV RNA were then tested individually, with repeatedly HIV RNA-reactive individual specimens declared positive. Specimens that were reactive on screening assay but non-reactive or inconclusive on confirmatory assay were tested individually for HIV RNA. The Abbott assay was also used for the additional research testing described below.

### Follow-up

All programs returned complete results at a follow-up visit. Patients from all programs who had positive HIV RNA results but negative or indeterminate antibody testing results were counseled that they had possible acute HIV infection and were asked to submit an additional specimen for confirmation of their status and HIV RNA quantitation.

### Study definitions of HIV infection

HIV status was only assigned for this study after all testing on all specimens (including re-testing on initially antibody-negative or discrepant specimens):

*HIV infection* was defined by having repeatedly reactive screening and confirmatory (WB or IFA) HIV antibody test results, *or* by having results that were repeatedly reactive for HIV RNA.

*Acute HIV infection* was defined by RNA-positive specimens having a negative or indeterminate pattern on the HIV antibody WB.

*Established HIV infection* was defined by antibody-positive specimens (on initial screening and confirmatory tests), or RNA-positive specimens having a reactive IFA or HIV antibody WB pattern that was positive [17].

### Analysis of assay performance (observed program data)

For each assay and specimen type used in clinical testing, we calculated performance parameters (sensitivity, specificity and positive and negative predictive values), with true HIV status defined by the reference standard of all detectable HIV infections, using the combined antibody plus HIV RNA algorithm as above. Where a single assay was used by multiple programs, data from all programs were combined for this analysis.

### Analysis of potential case finding

Because the testing population differed for each screening test in this first analysis, and because only four screening tests had been used in the field, we performed a second analysis allowing head to head comparison of a range of additional assays for detection of acute infections in the San Francisco testing population, using stored remnants of blood plasma:

#### Research testing on the “initially ab-negative or discrepant specimen panel

Any plasma specimens with discrepant, inconclusive, indeterminate, or negative antibody results but detectable HIV RNA were stored at −70°C for further analysis. The resulting panel of specimens was de-identified and submitted to the tests listed in Table 3. These included four commercially available, HIV antibody-only rapid tests; an HIV antibody Western blot (WB) assay, one whole viral lysate with recombinant antigen, IgG-sensitive (“2^nd^ generation”) antibody-only immunoassay, and one IgM-sensitive (“3^rd^ generation”) antibody-only immunoassay. Two newer assays, which at the time of this analysis were only available for sale outside the U.S., were also evaluated using this panel of archived specimens—the Determine® HIV-1/2 Ag/Ab Combo (Inverness Medical Innovations, Inc, Waltham, MA) and the ARCHITECT® HIV Ag/Ab Combo (List: 4J27, Abbott Diagnostics; Wiesbaden, Germany), a laboratory-based assay. Tests with visual readouts were read by two experienced public health microbiologists, with discrepant results resolved by a third reader.

**Table 3:** Ability of Tests to Detect Acute HIV Cases, and All HIV Cases: San Francisco Targeted Testing Programs, 2003–2008.

Test Name	Type	Format	Detection of HIV infection among Ab-/RNA+specimens (n = 66 cases)				Estimate of potential for case finding by each test	
			*Ab Screening Assay-, WB-/ind Acute Specimens (n = 58)*		*Ab Screening Assay- but WB+Specimens (n = 8)*		*Numerator includes 695 Ab screening test+, WB+specimens*	
			Test+	%	Test+	%	Test+	% (95 CI)
ARCHITECT HIV Ag/Ab Combo	Ag-Ab	Rapid lab IA	48/55	87.3	6/6	100.0	754/761	99.1 (91.1, 100.0)
Determine HIV-1/2 Ag/Ab Combo	Ag-Ab	POC RT	31/57	54.4	8/8	100.0	735/761	96.6 (84.7, 100.0)
			18 Ab+	31.5				
			13 Ag-only+	22.8				
Genetic Systems HIV-1/2 Plus O	Ab	Lab IA	20/58	34.5	8/8	100.0	723/761	95.0 (83.1, 100.0)
Unigold Recombigen HIV-1	Ab	POC RT	14/54	25.9	8/8	100.0	718/761	94.3 (82.4, 100.0)
Multispot HIV ½	Ab	POC RT	11/58	19.0	8/8	100.0	714/761	93.8 (82.9, 100.0)
Oraquick Advance (on blood plasma)*	Ab	POC RT	3/58	5.2	8/8	100.0	706/761	92.8 (88.2, 97.3)
Clearview Stat-Pak	Ab	POC RT	3/58	5.2	8/8	100.0	706/761	92.8 (83.8,100.0)
Vironostika HIV-1 Microelisa System	Ab	Lab IA	0/30	0.0	8/8	100.0	703/761	92.4 (89.0, 95.8)
rLAV HIV 1/2	Ab	Lab IA	0/24	0.0	6/8	75.0	701/761	92.1 (78.6, 100.0)
Genetic Systems HIV WB (any bands positive)	Ab	Western blot	16/58	27.6	8/8	100.0	719/761	94.4 (87.9, 100.0)

= immunoassay. RT = rapid test. POC = point-of-care. For each test, its potential for case finding was calculated by assuming it would detect the 695 specimens which were positive by the original HIV antibody screening assay, and also fully positive on supplemental Western blot testing. These estimates thus represent the maximum clinical sensitivity that could be expected were the assay to be used in San Francisco. For specimens that were negative in the original screening assay but reactive on pooled RNA testing, the ability of each test to detect these specimens was calculated separately for the 58 Western blot-negative or -indeterminate and for the 8 Western blot positive specimens, based on the test's observed performance on specimens tested in that category. When not all specimens in a category were available for testing, the proportion of tested cases detected was multiplied by the total number of cases in the category to obtain the estimate of potential case finding. IA

“acute” specimens that were reactive on the blood plasma sample, two had been negative on a screening test of fingerstick whole blood, using the same Oraquick Advance test. Actual performance estimates for Oraquick fingerstick blood testing are reported in Table 2. among the three

#### Estimating the potential impact of newer tests on HIV case finding

We used data from testing of the combined panel of initially Ab-negative or discrepant specimens to assess the potential of newer assays to detect additional cases, compared with those previously used for antibody screening. Specimens in the panel were categorized as acute HIV specimens that were Western blot negative or indeterminate (n = 58); specimens that were fully Western blot positive on re-testing (n = 8) were categorized as established HIV specimens (i.e., specimens that had been false-negative on initial testing). We estimated the number of additional acute or established cases that each assay could have detected, by multiplying the proportion of acute specimens and proportion of established specimens detected by each assay separately, and multiplying that proportion by the total number of specimens in each category in the testing population. To estimate how these cases would add to case finding overall in San Francisco, these case totals were added to the 695 cases of HIV that were already positive on initial *screening and confirmatory* antibody testing, and therefore not included in the panel.

## Results

### Acute and established HIV infections in testing populations

The targeted public health HIV testing programs included in this study tested 21,234 HIV patients during the study period. The combination of HIV RNA and HIV antibody tests identified 58 acute and 703 established HIV infections. As illustrated in Table 1, observed rates of acute and established infection varied significantly within the city of San Francisco, by testing program. In most of the targeted testing programs considered, acute infections constituted between 6 and 10 percent of all HIV infections detected. Among people requesting nPEP, however, 40.0 percent (8 of 20) HIV infections detected were acute infections at their first positive test. No acute infections were detected among sex workers. In addition to these SFDPH outcomes, the UCSF Optons study (a research-oriented testing program) identified 845 infections, among which 10.7 percent (90 of 845) were considered acute.

### Observed assay performance: clinical performance of blood and oral fluid tests compared with the antibody-RNA gold standard

Point estimates for assay sensitivity, specificity, and positive and negative predictive values were directly calculated for the four assays which were used for initial antibody testing (Table 2). Patients screened with blood specimens had observed test sensitivities of 92.3 percent (for the Vironostika 1^st^ generation antibody immunoassay), 91.9 percent (for the Oraquick Advance rapid test) and 96.2 percent (for the Genetic Systems 3^rd^ generation antibody immunoassay), compared with the antibody-plus-RNA reference standard. Estimated negative predictive values were excellent (>99.2 percent) for all of the screening assays, consistent with the fact that HIV is a low prevalence disease.

Among patients screened for HIV using oral fluid samples tested by the Oraquick Advance rapid test, only 86.6 percent (110/127) of HIV infections were detected, demonstrating a substantially lower sensitivity than any blood test. Of the six non-acute (i.e., blood antibody-positive) missed by oral fluid testing, all were detected by the Oraquick Advance device on the corresponding venous blood sample. Used on oral fluids, the Oraquick Advance test also had a slightly lower specificity than other tests, which resulted in a significantly lower positive predictive value (95.7 percent) for this oral fluid screening test than for any of the blood tests.

### Research testing to model the impact of test choice on potential for acute case finding in San Francisco

We estimated how each candidate assay would perform in acute case detection based on ability to detect infection in specimens, combined from all testing programs, that were *negative or indeterminate on a screening antibody test or on supplemental Western blot*. These estimates are given in Table 3. The ARCHITECT antigen-antibody combo immunoassay detected 87.4 percent of acute cases; the Determine antigen-antibody combo rapid test detected 54.4 percent; and the Unigold antibody-only rapid test detected 25.9 percent of the acute cases in this specimen panel. The most sensitive antibody-only test, the Genetic Systems 3^rd^-generation laboratory immunoassay, detected 34.5 percent of acute cases represented in the specimen panel.

Differences in detection of acute infections for the ARCHITECT combo immunoassay and Determine combo rapid test were related to the viral loads, measured on the same samples. Plasma viremia levels in acute infection specimens were in general extremely high, with a median of >500,000 and range of 77 to 500,000) copies HIV RNA/ml. While the ARCHITECT detected HIV infection in all antibody-negative specimens harboring greater than 12,183 copies/ml HIV RNA, the Determine detected no specimen with <500,000 copies/ml HIV RNA. Considering only the subset of 35 acute specimens that were negative on the “antibody” line of the combo rapid test device, 13 (37.4 percent) of these were positive on the “antigen” line.

For the two 4^th^ -generation combo assays used for experimental data, a panel of 81 HIV antibody- and RNA-negative specimens was used for preliminary assessment of specificity. The ARCHITECT combo immunoassay correctly identified 81 of 81 HIV negative specimens (a specificity of 100.0 percent (95.6, 100.0)) while the Determine combo rapid test correctly identified 80 of 81 HIV negative specimens, with 1 false positive result (a specificity of 98.8 percent (93.4, 99.7)). The one specimen falsely read as positive by our laboratory based on concordant readings by two microbiologists, exhibited a faint signal on the “antigen” band of the rapid test device.

### Impact of test choice on potential for overall case finding in San Francisco

**Figure 2:**
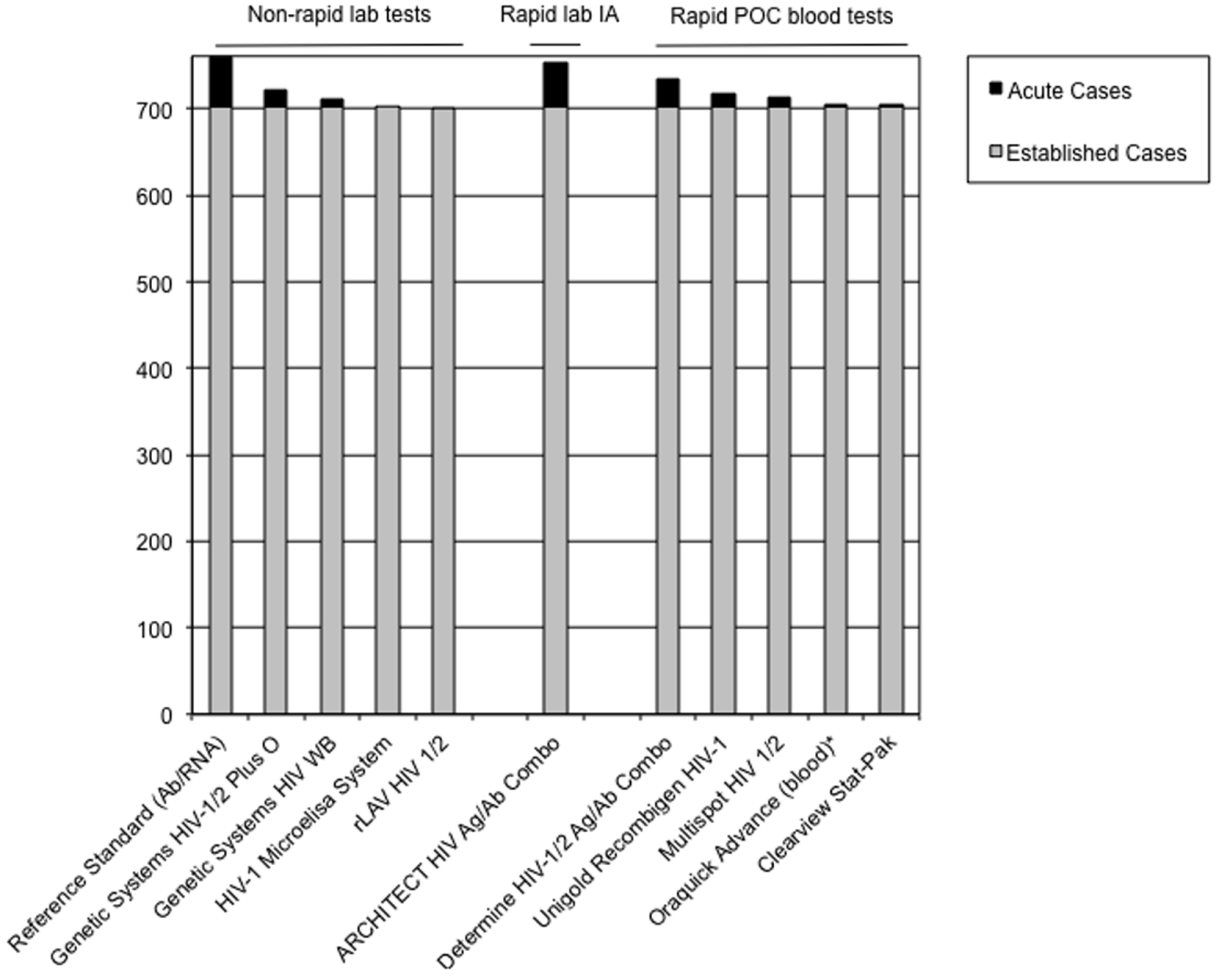
Estimated Impact of Test Performance on Detection of Acute (black) and Established (grey) HIV Infection Cases in San Francisco Targeted Testing Programs. For all blood assays, figures shown represent results shown in Table 3. For the Oraquick Advance assay, oral fluid results (Table 2) are not shown.

In a second analysis, we used the data on detection of the combined acute and established infection specimens in the panel to estimate how acute case detection by newer assays could have impacted overall HIV case detection had they been used in San Francisco. The potential for overall case detection was estimated at 99.1 percent for ARCHITECT combo, 96.9 percent for Determine combo, 94.3 percent for Unigold and somewhat lower for Multispot (93.8), StatPak (92.8) or blood-based Oraquick Advance (92.8) antibody-only rapid tests. These estimates are shown in Table 3, and illustrated in Figure 2. For three blood assays (Oraquick, Vironostika and Genetic Systems) that were used in clinical testing and also used to re-test the panel of stored specimens, we found that the panel based estimates of potential case finding were similar to observed performance.

## Discussion

In this study, we confirmed that differences between HIV tests in current use translate to substantial differences in case identification in real world HIV testing practice. We focused particularly on the capabilities of newer technologies that are capable of delivering rapid HIV test results and are therefore especially useful in drop-in testing sites and in medical settings where rapid results are needed to guide patient management [17]. In two separate analyses that each pitted tests against a gold standard involving both HIV antibody and HIV RNA testing [6], [7], [20]–[22] we found that the range of “rapid tests” now available vary significantly in their ability to detect acute HIV cases. In one analysis, we analyzed the potential performance of two newer tests developed to detect both HIV antibodies and HIV p24 antigen (“4^th^ generation” or “Ag-Ab combo” tests): the first, a 4^th^ generation immunoassay (which, while not a point-of-care test, can give rapid results in random access mode) identified 87 percent of acute HIV cases. A second, developmental point-of-care 4^th^ generation combo test detected 54 percent of acute cases in this same analysis.

These combo tests also have sensitivity for HIV antibodies. Because of high rates of acute case detection, we estimated in this study that the rapid laboratory immunoassay could detect 99 percent of all cases detected by the much more costly and complex antibody test-plus-RNA algorithm. This result supports a recommendation that all laboratories for high risk HIV testing programs that currently use antibody-only immunoassays should consider using a 4^th^ generation, antigen-antibody combo test (see [23]). The ability to obtain rapid results with an automated 4^th^ generation immunoassay also suggests that this or similar tests could be more broadly deployed for rapid screening for acute HIV in acute care medical settings—settings where HIV testing is rarely done [17]–[19] but where acute infections may be surprisingly prevalent [24]–[26].

Based on performance seen in testing stored blood plasma specimens, we estimated that the one developmental “4^th^ generation” rapid test (Determine HIV HIV 1/2 Ag-Ab Combo) we examined in this study could have detected as many as 97 percent of all cases. This estimate was better than any other point-of-care rapid blood test we examined. The antibody line on the test strip was quite sensitive, reading positive for early antibodies in 18 of the 57 Western-blot negative or indeterminate infections in our testing population. Among the remaining 35 acute infection specimens missed by the antibody line, the p24 antigen line gave a (sometimes faint) positive signal in 13 acute infection cases. Of note, all of the 13 antigen-only positive acute cases had viral loads in excess of 500,000 copies/ml HIV RNA. The ability of the Determine combo device to detect antibody-negative acute infections was notably better than has been seen in previous, smaller-scale clinical studies of this same test [27], [28]. Reasons for this are unclear but might include use of blood plasma (rather than whole blood) for the research testing in this study. Additional field studies may be warranted.

We were concerned by the relatively poor clinical performance of the one approved device for testing oral fluid for HIV antibodies (the Oraquick Advance test)—which has recently been approved for home use in settings where risk may be high. Oral fluid testing the had clinical sensitivity of only 87 percent—missing as many as one in six HIV infections that could be detected by blood testing. Importantly, the test failed to detect *both acute infections and a smaller number of antibody-positive established infections*. In addition to the risk of false-positives that has been well described with oral fluid testing [29]–[32], our study suggests that such oral fluid testing can pose a real risk of providing false-negative results to HIV-infected patients. These concerns must therefore be carefully weighed against the potential advantages of home based testing or oral fluid testing when choosing a testing strategy for individuals at high-risk of HIV infection.

The Oraquick test performed much better when used to test fingerstick blood, and its performance in research testing, on stored blood plasma specimens, was similar to that for other rapid antibody tests.

An important concern with the use of the newer “4^th^ generation” combo assays for HIV screening is that each can return a relatively large proportion of screening test-positive, confirmatory antibody test-negative results, since initial supplemental/confirmation testing is restricted to supplemental antibody assays. HIV RNA testing on the initial sample is recommended to help establish true HIV status in patients with such discrepant results [12], [16].

Several limitations must be considered in interpreting the results of these analyses. First, the five antibody screening tests were compared against a common reference standard, but they were used in slightly different populations and at different times; hence, assay performance comparisons are not strictly head-to-head. All populations were combined for the second analysis, and a larger number of tests were evaluated in a head to head fashion. However, for this analysis we were only able to test those specimens that had been archived because of initially discrepant clinical test results. By seeing which of these ‘problem specimens’ were detected by each of the newer tests, we could estimate the potential for additional case finding that could be expected using the newer tests. The resulting estimates of potential case finding corresponded very closely to the actual clinical sensitivity estimates that were available for three blood assays. For this reason we believe that our estimates of potential case finding are very close approximations of the true clinical sensitivity for the tests evaluated in this study. Third, our results were based on testing plasma and may not be generalizable to how some assays may perform in testing finger-stick whole blood. Fourth, the study design precluded a rigorous analysis of specificity of the 4^th^ generation combo assays used in this study. A multicenter, prospective trial comparing assay performance for an FDA-approved version of the ARCHITECT HIV Ag/Ab immunoassay against a pooled RNA gold standard is now underway. Finally, the conclusions of our study are not necessarily generalizable to all HIV screening situations. In programs for expanded, ‘routine’ HIV screening in which the prevalence of acute HIV infections is known to be very low, currently approved HIV rapid tests and immunoassays could be expected to have excellent sensitivity.

For targeted HIV testing programs in high-risk settings like San Francisco, this study demonstrates the extent to which the selection of an HIV screening assay can directly impact HIV case finding in general, and acute HIV case finding in particular. Results suggest that the availability of newer, 4^th^ generation combo immunoassays or point-of-care rapid tests could represent a major advance for HIV diagnostics and prevention by permitting much more rapid testing for acute HIV infection in diverse clinical settings.
